# Newly Found Peacekeeper: Potential of CD8+ Tregs for Graft-Versus-Host Disease

**DOI:** 10.3389/fimmu.2021.764786

**Published:** 2021-11-24

**Authors:** Weihao Wang, Tao Hong, Xiaoqi Wang, Rui Wang, Yuxuan Du, Qiangguo Gao, Shijie Yang, Xi Zhang

**Affiliations:** ^1^ Medical Center of Hematology, Xinqiao Hospital, Army Medical University, Chongqing, China; ^2^ State Key Laboratory of Trauma, Burns and Combined Injury, Army Medical University, Chongqing, China; ^3^ Department of Laboratory Medicine, the Affiliated Hospital of Zunyi Medical University, Zunyi, China; ^4^ Department of Cell Biology, College of Basic Medicine, Army Medical University, Chongqing, China

**Keywords:** hematopoietic stem cell transplantation, graft-versus-host disease, CD8, CD4, regulatory T cells

## Abstract

Allogeneic hematopoietic stem cell transplantation (allo-HSCT) remains the most effective and potentially curative treatment for a variety of hematologic malignancies. However, graft-versus-host disease (GVHD) is a major obstacle that limits wide application of allo-HSCT, despite the development of prophylactic strategies. Owing to experimental and clinical advances in the field, GVHD is characterized by disruption of the balance between effector and regulatory immune cells, resulting in higher inflammatory cytokine levels. A reduction in regulatory T cells (Tregs) has been associated with limiting recalibration of inflammatory overaction and maintaining immune tolerance. Moreover, accumulating evidence suggests that immunoregulation may be useful for preventing GVHD. As opposed to CD4^+^ Tregs, the CD8^+^ Tregs population, which constitutes an important proportion of all Tregs, efficiently attenuates GVHD while sparing graft-versus-leukemic (GVL) effects. CD8^+^ Tregs may provide another form of cellular therapy for preventing GVHD and preserving GVL effects, and understanding the underlying mechanisms that different from those of CD4^+^ Tregs is significant. In this review, we summarize preclinical experiments that have demonstrated the role of CD8^+^ Tregs during GVHD and attempted to obtain optimized CD8^+^ Tregs. Notably, although optimized CD8^+^ Tregs have obvious advantages, more exploration is needed to determine how to apply them in the clinic.

## 1 Introduction

Allo-HSCT was established to treat patients with hematological malignancies. Rejection, GVHD and infections are major clinical complications after allo-HSCT and are associated with graft failure and transplant-related morbidity and mortality. These complications continue to impede successful transplantation and limit its curative effect. Thus, overcoming such complications is the main objective to improve allo-HSCT outcomes and to patient quality of life. Preventing GVHD with immunosuppressant may lead to side effects such as severe infection, hematologic malignancy relapse and multiorgan dysfunction, which are important factors in transplantation-related mortality. In addition, broad- and long-term immunosuppressive therapy for GVHD may subsequently dampen beneficial GVL responses, constituting an ongoing challenge.

Researchers have discovered several regulatory cell subsets that prevent the occurrence and development of GVHD, among which adoptive regulatory Tregs suppress the function of effector T cells (1) and play a crucial role in limiting immune response overaction, regulating immune homeostasis (2) and maintaining tolerance (3), improving the outcome of GVHD. In addition to CD4^+^ Tregs, which dominate regulatory cells in autoimmune diseases, CD8^+^ suppressor T cells are emerging as an important subset of regulatory T cells and have received much attention. Recently, more and more clinical data have verified the significant roles. Researchers have found a lower proportion of CD8^+^ Tregs in GVHD patients (4). Moreover, patients with autoimmune diseases like multiple sclerosis (5), type 1 diabetes (6), common variable immunodeficiency (7) have shown the same tendency of CD8^+^ Tregs. In addition, patients after HSCT with lupus remission have a greater number of CD8^+^ Tregs (8) and anti-CD20 treatment in multiple sclerosis is also associated with increased CD8^+^ Tregs (9), indicating that CD8^+^ Tregs could alleviate immune response. However, CD8^+^ Tregs do not always benefit to health, which showed higher proportions in chronic lymphocytic leukemia (10), multiple myeloma (11) and solid tumors like hepatocellular carcinoma, ovarian cancer and lung cancer (12–14). But alloreactive CD8^+^ Tregs are less stable than CD4^+^ Tregs but efficiently attenuate GVHD while preserving the GVL effect. Although CD8^+^ Tregs were described before CD4^+^ Tregs, their biology is less well understood due in part to their small numbers, thus rendering functional studies difficult.

In this review, we discuss the biology and development of CD8^+^ Tregs, their contribution to reducing GVHD while preserving GVL effects apart from CD4^+^ Tregs as well as their heterogeneity, focusing on questions and future improvements through genetic technologies.

## 2 Revival of CD8^+^ Tregs

CD8^+^ Tregs were the first suppressive cells reported (15). Despite many studies, the exact definition of CD8^+^ Tregs remains unclear, and a lack of assessment has caused difficulty in marker discovery. Expression of the common and sensitive marker FOXP3 is lower in CD8^+^ Tregs than in CD4^+^ Tregs in both mouse and human studies. However, FOXP3 correlate with the activation and potential suppressive capacity of CD8^+^ Tregs (16). A study in 2018 involving CD4^+^ Tregs single-cell RNA sequencing highlighted some important transcription factors and membrane molecules related to immunosuppressive functions, including FOXP3, IKZF2 (IKAROS), TNFR2, IL2RA and IL2RB, in mice and humans (17). Recent data on CD8^+^ Tregs confirm that Bim and Mcl-1 expression contributes to immune regulation (18), though the regulatory ability of Tregs subsets differ. Studies have demonstrated that CD4^+^ Tregs function correlates with the repertoire of TCRs (19, 20), whereas CD8^+^ Tregs are largely related to the proapoptotic phenotype (18). CD8^+^ Tregs mainly interact with CD8^+^ T cells, which is different from the interaction and collaboration of CD4^+^ Tregs and CD4^+^ T cells and the collaboration between them really truly exists (21), yet several studies suggest that CD8^+^ Tregs act on CD4^+^ T cells or both CD4^+^ and CD8^+^ T cells (22, 23). For CD8^+^ Tregs, anti-PD-1 can only block suppression of CD8^+^ T cells, but has no effect on CD4^+^ T cells, even though the combination of anti-PD-1 and anti-CTLA-4 completely abrogates the suppressive effect on CD4^+^ T cells (24). We speculate that CD8^+^ Tregs may affect both CD4^+^ and CD8^+^ T cells in different ways, and the specific signaling pathways hence need to be clarified.

During the GVHD process, CD8^+^ Tregs are more remarkable than CD4^+^ Tregs to some extent, constituting a suppressive population and attenuating the severity of this disease (25). Prior studies have shown that not only does the proliferation of CD8^+^ Tregs exceed that of CD4^+^ Tregs under certain conditions (16, 25), but the induced CD8^+^ Tregs (CD8^+^ iTregs) represent a larger proportion of induced Tregs (iTregs) in GVHD (26), which implies that it is easier to induce the formation of CD8^+^ iTregs *in vitro* and may help to address the insufficient source of CD4^+^ Tregs. In addition, the generation of CD8^+^ Tregs only occurs in the presence of allogeneic MHC (26) and correlates positively with the level of MHC disparity (25, 27). Further studies have shown that the antigen-specific regulatory abilities related to MHC-I only involve CD8^+^ Tregs (28). Importantly, CD8^+^ iTregs conditionally suppress allogeneic reactions without impairing general immunity against pathogen infection and residual tumor recurrence, in contrast to unselective CD4^+^ Tregs (29). Nevertheless, the CD8^hi^ Tregs induced by CD40-activated B cells (30) and CD4^+^ Tregs induced by allogeneic antigens *in vitro* (21) are both antigen-specific. We speculate that the proportion of CD4^+^ iTregs decides whether CD4^+^ Tregs are antigen-specific and such conflicting results may derive from the fact that there is a larger proportion of natural occurring Tregs (nTregs) in CD4^+^ Tregs populations during GVHD, which present less targeted immunoregulation. There has been significant progress in our understanding of the immune regulation ability of CD8^+^ Tregs, becoming a hot topic for immune disorders.

## 3 CD8^+^ Tregs Alleviating GVHD While Preserving GVL Effects

**Figure 1:**
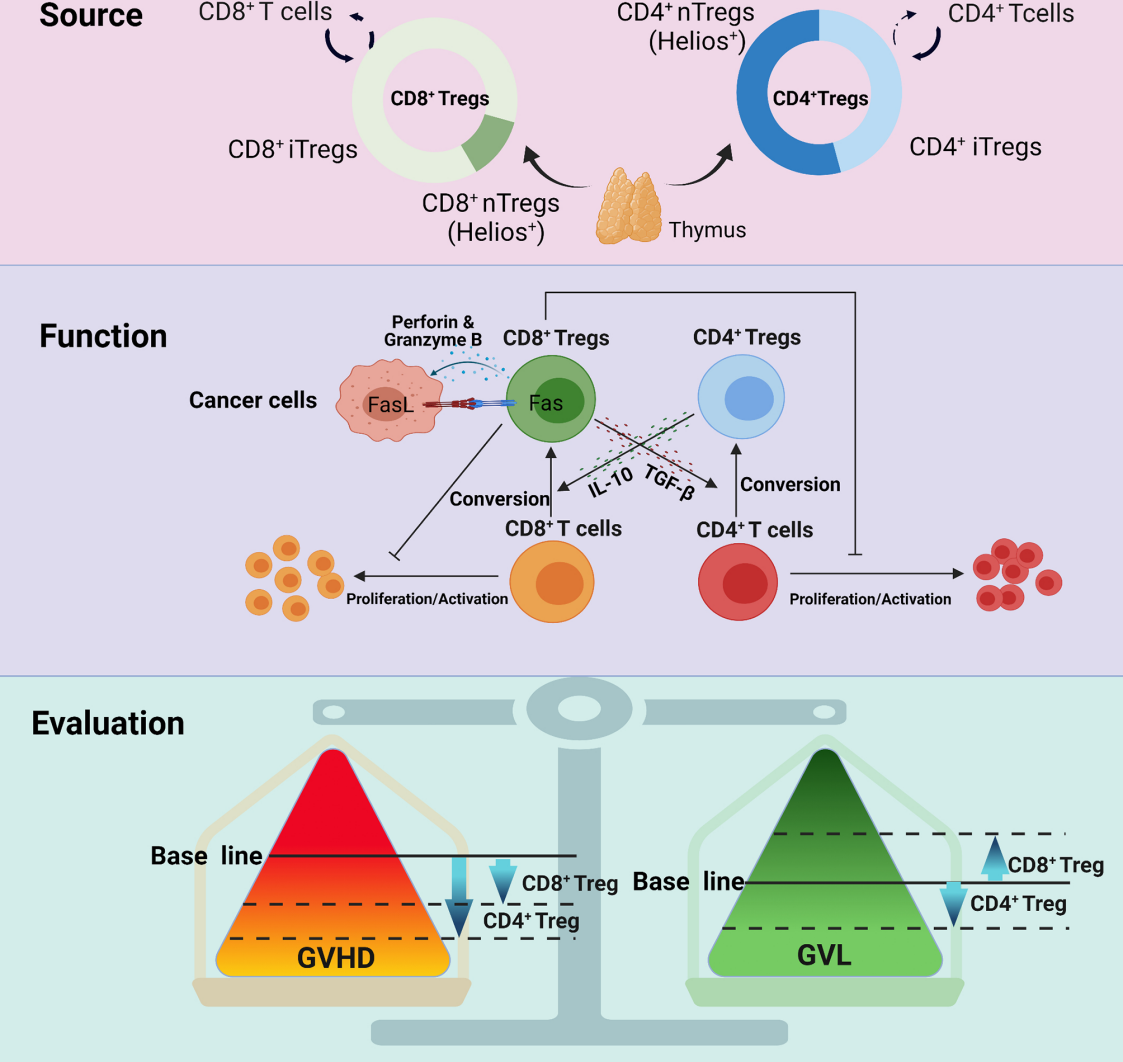
The different manifestations between CD8^+^ Tregs and CD4^+^ Tregs with regard to source, function and evaluation. CD8^+^ Tregs mainly derive from CD8^+^ T cells and rarely from the thymus; CD4^+^ Tregs originate from both sources. CD8^+^ Tregs secrete cytokines, such as IL-10 and TGF-β, and exert an influence on GVHD, but their GVL effect mainly depends on the Fas-Fasl interaction, perforin and granzyme B This may be the reason why CD8^+^ Tregs retain GVL but that CD4^+^ Tregs partially impair GVL. Studies also indicate that CD4^+^ Tregs are more powerful in regulating GVHD.

Unlike CD4^+^ Tregs, the mechanisms by which CD8^+^ Tregs alleviate GVHD are not entirely clear. Furthermore, growing evidence support the maintenance of GVL effect for CD8^+^ Tregs. Here, we summarize the regulatory mechanisms of CD8^+^ Tregs in GVHD and GVL effects (**Figure 1**).

### 3.1 Initiation of CD8^+^ Tregs in GVHD

A previous study illustrated that CD8^+^Foxp3^-^ T cells can convert to CD8^+^Foxp3^+^ Tregs with the help of DCs and TGF-β from hosts in mesenteric lymph nodes after allo-HSCT (28). Gut-associated DCs induce CD8^+^ Tregs, depending on TGF-β and retinoic acid (RA) (31). Therefore, antigen-presenting cells (APCs) may initiate induction, with cytokines assisting in the process. The CD8^+^ iTregs largely migrate and proliferate into the gastrointestinal tract and spleen, and the instability of Fxop3 will eventually result in conversion to effector cells, leading to GVHD (32). For skin allografts, CD8^+^ Tregs would mainly gather in draining lymph nodes (33). In summary, CD8^+^Foxp3^+^ Tregs preferentially migrate to the site of immunoreaction; and communication with the local microenvironment is highly important, including the response to cytokines and cell contact.

### 3.2 Intercellular Interactions of CD8^+^ Tregs in GVHD

In GVHD, CD8^+^Foxp3^+^ Tregs exert protective effects principally by inhibiting CD4^+^ T cells and B cells, or including CD8^+^ T cells (22, 23). However, there are inconsistent results indicating that CD8^+^ Tregs significantly suppress CD8^+^ T cells instead of CD4^+^ T cells (21). The contradictory results come from different measurement indexs and show discrepant effects in different aspects, which needs further study to acquire a credible answer. In the humanized GVHD mouse model, CD8^+^ Tregs suppress T cell proliferation and inflammatory cytokine factor release for a long time in CTLA-4 (34) and PD-1 (24) dependent manners. In addition to T cell suppression, CD8^+^Foxp3^+^ Tregs induce CD4^+^ Tregs *in vivo* in a manner dependent on TGF-β secretion by CD8^+^Foxp3^+^ Tregs (33). Subsequent experiments show that CD8^+^ Tregs induce both CD8^+^ and CD4^+^ Tregs in a GVHD model (22). CD8^+^CCR7^+^ Tregs coexpressing CD45RA, Foxp3 and CD28 exert regulatory functions by suppressing TCR signal-mediated phosphorylation of ZAP70 in CD4^+^ T cells, the initial step of T cell activation, and decreasing intracellular calcium signaling (35). Phosphorylation of ZAP70 has also been found to play an important role in GVHD (36). Many results indicate that CD8^+^ Tregs is closely linked to DCs. CD8^+^Foxp3^+^ Tregs actually react with DCs by decreasing the expression of CD40 and CD80/CD86, which also indicates that CD8^+^Foxp3^+^ Tregs contribute to the immune reactions (28, 33). Evidence demonstrates that CD8^+^ Tregs become conditioned to react with plasmacytoid DCs but not conventional DCs (16). However, whether discrepant responses influence their regulatory abilities regarding GVHD development *in vivo* remains unclear. CD8^+^ Tregs participate in other mechanisms, such as modulating Th17 (37), though more direct evidence for relevance to GVDH is needed.

### 3.3 IL-10 and TGF-β Immunoregulation in CD8^+^ Tregs

Among all regulatory factors, IL-10 and TGF-β attract the most attention. As analyzed in human serum, IL-10 and TGF-β exhibit a negative correlation with the occurrence of GVHD (38), which supports their regulatory roles. *In vitro*, CD8^+^ Tregs perform regulatory functions on allogeneic antigens in a fashion extremely dependent on IL-10 and TGF-β but not cytotoxicity (30). In lupus patients, CD8^+^Foxp3^+^ Tregs play a regulatory role dependent on TGF-β after allo-HSCT, with no contact with CD4^+^ T cells (8). Similarly, IL-10 possesses strong regulatory abilities, and TGF-β is significant for maintaining CD8^+^ Tregs (39). However, *ex vivo*-induced CD8^hi^ Tregs decrease expression of IL-10 and TGF-β in a humanized GVHD mouse model, which does not negate their contribution to GVHD (34). Researchers have also found that CD8^+^Foxp3^+^ Tregs produce minor amounts of IL-10, ruling out toxicity in regulatory capacity (28). In conclusion, IL-10 and TGF-β are important for CD8^+^Foxp3^+^ Tregs, even though their expression is decreased. CD8^+^CD103^+^ Tregs also depend on IL-10 and TGF-β and show no relationship with cytotoxicity in an autoimmune disease model (40). In a cGVHD lupus mouse model, CD8^+^CD103^+^ Tregs effectively alleviate the severity of GVHD, demonstrating the dependence on TGF-β and IL-10 without any cytotoxicity, with cell contact being indispensable (41). However, it has also been reported that human alloantigen-induced CD8^+^CD103^+^ Tregs are independent on IL-10 and TGF-β *in vitro* as well as cytotoxicity (42). Overall, the inconsequent expression and dependence of IL-10 and TGF-β and cytotoxicity in CD8^+^ Tregs remain uncertain and may be associated with different induction methods or complex inflammatory microenvironments.

### 3.4 CD8^+^ Tregs in Preserving GVL Effects

Mature donor T cells found in the graft become allo-activated during allo-HSCT, which leads to not only T cell proliferation and migration to target organs but also to activation for clearance of residual malignant cells, which is GVL effects (43). To date, it has been very difficult to separate GVHD and GVL effects. It is commonly reported that CD4^+^ Tregs impair the GVL effect when alleviating GVHD. Surprisingly, CD8^+^ Tregs maintain and even exert the GVL effects (21, 23, 32), which is another distinctly superior aspect of CD8^+^ Tregs and indirectly reflects antigenic specificity. It has also been reported that CD8^+^ Tregs virtually exert GVL effects through the Fas-FasL and perforin–granzyme B pathways without affecting general immunity but that CD4^+^ Tregs do not (34), which is consistent with results that the CD8^+^ iTregs retain cytotoxicity and preserve the GVL effect (22). Further results demonstrate that the cytotoxicity of CD8^+^ Tregs in tumor cell killing is the basis of GVL effects and that the combination of CD4^+^ Tregs and CD8^+^ Tregs is an ideal therapy that would more effectively prevent GVHD and preserve GVL effects (21).

## 4 CD8^+^ Tregs Heterogeneity

In general, the CD4^+^ Tregs phenotype is identified, as CD4^+^CD25^high^Foxp3^+^ and/or CD127^low/-^ and Foxp3 is necessary for maintaining regulatory functions, but CD8^+^ Tregs display much more heterogeneity in phenotype and function (44, 45). There are diverse types of CD8^+^ Tregs for different disease conditions, presenting various effects (33, 46–48). (**Table 1**) Several CD8^+^ Tregs could naturally occur and participate in normal living activities. And researcher have found that there are abnormal CD8^+^ Tregs in several circumstances, including decreasing in GVHD and autoimmune disease patients, while elevating in patients with cancers. But some other CD8^+^ Tregs such as CD8^+^CD103^+^ Tregs could be cultured *in vitro* and infused back for disease therapy.

**Table 1:** Heterogeneity of CD8^+^ Tregs.

Phenotype	Features	Ways to acquire	Source	Effect in GVHD	Ref.
CD8^+^CD25^+^Foxp3^+^	Expressing CTLA-4, TNFR2 and other various surface makers for different situations such as GITR, CD44, CD102 and CD133, dependent on IL-2 and TGF-β for phenotype and function	Naturally occurring and induced	Humans and Mice	Yes	(25, 26, 28, 29, 31, 33, 39, 49–51)
CD8^+^CD103^+^	CD103 may compensate the deficiency of Foxp3 in regulating immune response, and dependent on IL-2 and TGF-β and is more stable than CD4^+^Tregs to some extent.	Induced	Humans and Mice	Yes	(40–42, 52, 53)
CD8^hi^ Tregs	Antigen-specific and expressing CD25, Foxp3 and CTLA-4 extremely similar to CD8^+^CD25^+^Foxp3^+^ Tregs	Induced	Humans	Yes	(30, 34, 54)
CD8^+^CD45RC^low/-^	Expressing foxp3 and dependent on production of IL-10 and TGF-β	Naturally occurring and induced	Humans and Rats	Yes	(16, 55)
CD8^+^CD28^-/low^	Less dependent on Foxp3 for immunoregulatory properties, and other molecules may be highly relevant	CD8^+^CD28^low^ Tregs are more likely to occur naturally, and CD8^+^CD28^-^ Tregs tend to be induced	Humans and Mice	not described	(56–59)
CD8^+^CD122^+^	Dependent on PD-1 and CD28 but not Foxp3, which is related to recognition of CD80/86 and production of IL-10; sometimes more powerful than CD4^+^ Tregs	Induced	Mice (human CD8^+^CXCR3^+^ Tregs may be the counterparts of mice CD8^+^CD122^+^ Tregs)	not described	(60–66)

### 4.1 CD8^+^CD25^+^Foxp3^+^ Tregs

CD25^+^ T cells have a unique regulatory function mainly *via* consuming endogenous IL-2. In contrast, previous studies have found that CD8^+^CD25^+^ T cells and CD4^+^CD25^+^ T cells play an active role in the GVHD process (67, 68), suggesting that CD25 is insufficient to identify CD8^+^ Tregs. The regulatory functions of CD4^+^ Tregs have been proven to be extremely dependent on Fxop3 (69), which is considered a regulatory marker for Tregs. CD8^+^CD25^+^Foxp3^+^ Tregs *in vivo* have similar regulatory ability and express CTLA-4 and TNFR2 (49), and induced CD8^+^CD25^+^Foxp3^+^ Tregs similarly express CTLA-4, PD-1, PD-L1, and TNFR2 *in vitro*. Furthermore, expression of PD-L1 and TNFR2 is imperative for regulatory functions, which are maintained by IL-2 and TGF-β (33, 39, 50). Most importantly, there is considerable evidence showing that CD8^+^CD25^+^Foxp3^+^ Tregs effectively regulate GVHD (25, 29, 51). Despite expressing GITR and CTLA-4 like CD4^+^ Tregs, CD8^+^CD25^+^Foxp3^+^ Tregs rarely express Helios regarded as a marker for nTregs from the thymus, which means that almost all CD8^+^CD25^+^Foxp3^+^ Tregs are induced to generate, relying on the receptors of IL-2 and TGF-β (26, 28).

### 4.2 CD8^+^CD103^+^ Tregs

CD103 is generally expressed on the surface of partial T cells and DCs and is related to tissue location as the receptor of E-cadherin. Experiments have identified that expression of CD103 on CD4^+^ Tregs contributes to local regulatory abilities (70) and that expression on effector CD8^+^ T cells aids in entry into and destruction of the intestinal epithelium in animal models of GVHD (71), which indirectly supports the same role for CD8^+^CD103^+^ Tregs. CD8^+^CD103^+^ Tregs usually derive from CD8^+^CD103^-^ T cells, and CD103 is regarded as the marker of CD8^+^CD103^+^ Tregs (42). The regulatory ability of CD8^+^ Tregs is independent of Foxp3; for CD8^+^Foxp3^-^ Tregs, CD103 is needed and seemingly remedies Foxp3 deficiency (40). CD103 expression induced by human cord blood mononuclear cell (CBMC) on CD8^+^ T cells has been proven, and culture conditions can drive differentiation of CD8^+^CD25^+^Foxp3^+^CD103^+^ Tregs from human CBMCs (52). During GVHD, CD8^+^CD103^+^ Tregs induced by TGF-β effectively alleviate disease severity; they are more stable than CD4^+^ Tregs because they express CD103 (41). CD39 also plays a regulatory role in CD8^+^CD103^+^ Tregs, and anti-CD39 abrogates regulatory abilities (53).

### 4.3 CD8^hi^ Tregs

CD8^hi^ Tregs induced by CD40-activated B cells are antigen-specific, and functional markers, such as CD25, Foxp3, CTLA-4, GITR, IL-10, and TGF-β, are upregulated (30). These findings are consistent with the results that CD8^hi^ Tregs induced *in vitro* with expression of CD25, Foxp3 and CTLA-4 alleviate GVHD without affecting general immunity and graft-versus-tumor activity (34). CD8^hi^ Tregs are also induced by human monocyte-derived suppressor cells (HuMDSCs) *in vivo*, showing upregulation of CD25, Foxp3 and CD103 (54). Despite plentiful evidence for the existence of CD8^hi^ Tregs, it is uncertain whether they are entirely different from CD8^+^CD25^+^Foxp3^+^ Tregs. Indeed, both express the phenotype analogously, and expression of CD25 and Foxp3 may be concomitant.

### 4.4 CD8^+^CD45RC^low/-^ Tregs

Natural CD8^+^CD45RC^low/-^ Tregs coexpressing Foxp3 and CTLA-4 indicate an immunosuppressive function (55). Similarly, CD8^+^CD45RC^low/-^ Tregs induced *in vitro* richly expressing Foxp3, CD25, CD103, CD122 and GITR have been identified to play a vital role in GVHD (16).

### 4.5 Other Subgroups of CD8^+^ Tregs

For some types of CD8^+^ Tregs, direct evidence to identify their immunomodulatory effects in GVHD is currently lacking.

#### 4.5.1 CD8^+^CD28^-/low^ Tregs

CD28 plays a key role as a costimulatory signal in adaptive immunity by activating T cells. However, a subset of CD8^+^ Tregs was recently identified as CD28^-^ or CD28^low^. In that study, CD8^+^CD28^-^ Tregs developed naturally *in vivo* with expression of CD25, Foxp3 and CTLA-4, along with low expression of CD127 and high expression of CD122 (56, 57). CD8^+^CD28^-^ Tregs can be induced *in vitro* without expression of Foxp3, whereas CD4^+^ Tregs cannot. GITR expression is crucial for their generation (58). CD39 (ectonucleoside triphosphate diphosphohydrolase 1; encoded by ENTPD1) binds extracellular ATP (eATP) and converts it to extracellular adenosine which is expressed by various immune cells and non-immune cells (72) and expression of CD39 plays an important role in CD8^+^CD28^-^ Tregs without Fxop3, CD103 and CD122 expression (59). Regarding the origin of CD8^+^CD28^-^ Tregs, researchers have suggested that they may derive from CD8^+^CD28^-^ T cells associated with IL-10 secretion but not from CD8^+^CD28^+^ T cells (73). Regardless, CD8^+^CD28^low^ Tregs are present in the thymus locally without recirculating from the periphery; they express Helios but not traditional CD4^+^ Treg markers such as Foxp3, CD25 and neuropilin-1 (74), and these cells tend to be generated from the thymus (57). In general, CD8^+^CD28^-^ Tregs and CD8^+^CD28^low^ Tregs differ from each other, and their regulatory capacities and application in GVHD should be studied further.

#### 4.5.2 CD8^+^CD122^+^ Tregs

CD122 is usually known as the beta chain of IL-2 or IL-15. Initially, transferred CD8^+^CD122^+^ T cells prevent the development of activated CD69^+^ T cells, indicating a single group of regulatory T cells (60). Subsequent experiments discovered the importance of PD-1 and CD28 for regulatory abilities, and CD8^+^CD122^+^PD-1^+^ T cells can be distinguished from CD8^+^CD122^+^ T cells (61). In addition, CD28 may help CD8^+^CD122^+^ Tregs recognize target cells by interacting with CD80/86, excluding the regulatory function of CTLA-4 and ICOS, but Foxp3 is not expressed in CD8^+^CD122^+^ Tregs (62). It is worth noting that previous studies have shown that the addition of PD-L1 enhances IL-10 expression, but another study indicated that anti-PD-1 was unable to inhibit IL-10. These discrepancies may be due to different induction methods, thus affecting the response to PD-1 signals. IL-10 expression is strongly reduced by blocking CD28, which indicates that the interaction between CD28 and CD80/86 is indispensable for regulatory abilities (63). Considering the discrepancy between humans and animals, CD8^+^CD122^+^ Tregs in mice correspond to CD8^+^CXCR3^+^ Tregs in humans (64). In allotransplant animal models, the regulatory abilities of CD8^+^CD122^+^ Tregs are superior to those of CD4^+^ Tregs in terms of the production of IL-10 (65). In addition, emodin can alleviate immunological rejections by inducing CD8^+^CD122^+^ Tregs and CD4^+^ Tregs *in vivo (* 66). However, the exact function of CD8^+^CD122^+^ Tregs in GVHD remains unclear, and further study is urgently needed.

## 5 CD8^+^ Tregs Optimization

**Figure 2:**
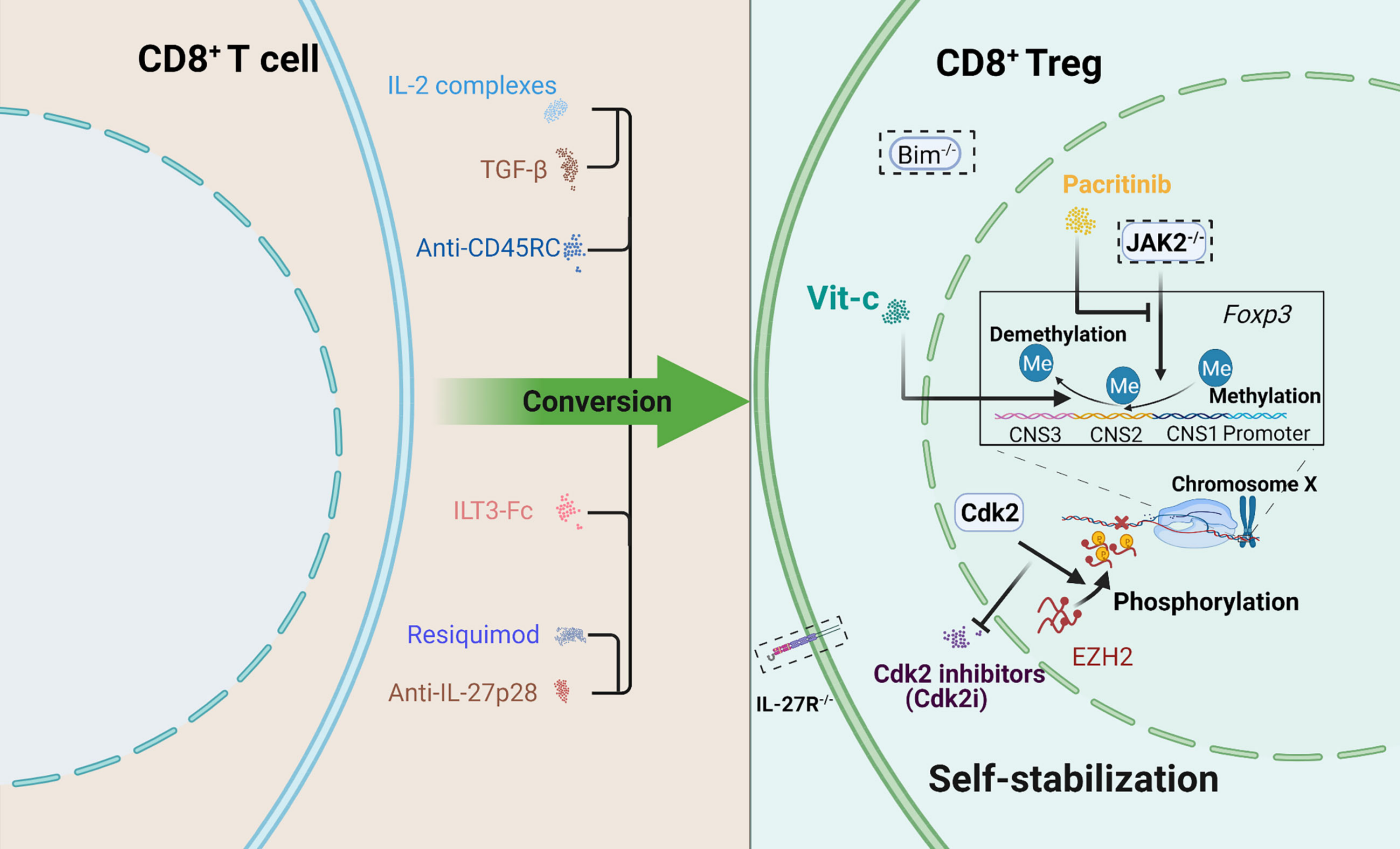
Schematic depicting several attempts to optimize CD8^+^ Tregs. Two aspects were included: promoting conversion and maintaining self-stabilization. How to maintain stable expression of Foxp3 is a priority. Some approaches offer new therapeutic ideas for the clinic. A dotted box indicates gene knockout.

Continuing research on CD8^+^ Tregs to support a stable state and enhanced regulatory ability is needed to attenuate GVHD effectively, which means the need for CD8^+^ Tregs optimization. In addition, ensuring safety is of much significance. Attempts for CD8^+^ Tregs improvement are provided in **Figure 2**.

### 5.1 Promoting Generation

Several cytokines have been described as playing a role in CD8^+^ Tregs. IL-2 is a proinflammatory factor, and recent studies have demonstrated its regulatory functions in GVHD. Expansion of CD4^+^ Tregs induced by IL-2 occurs during GVHD treatment (75, 76), and the ability of this cytokine to regulate CD8^+^ Tregs needs to be further studied. Rapamycin and IL-2 have been described as expanding CD8^+^Foxp3^+^ Tregs *in vivo*, contributing to GVHD severity alleviation (28). Consistently, rapamycin enhances the immunoregulatory properties of CD8^+^Foxp3^+^ Tregs (32). Rapamycin and IL-2 show a limited effect on the stability of CD8^+^Foxp3^+^ Tregs compared to CD4^+^ Tregs; thus, the former may not completely eradicate GVHD due to conversion into effector T cells (32). Moreover, CD8^+^CD45RC^low/-^ Tregs prevent GVHD *via* extensive regulatory functions and proliferation abilities induced by rapamycin (16). A study on all-trans retinoic acid (ATRA) suggested that it promotes Foxp3 demethylation to increase CD4^+^ Tregs production (77). In GVHD models, ATRA promotes CD4^+^ Tregs generation to effectively alleviate GVHD but is unfavorable for CD8^+^ Tregs (78), which in turn indicates a differential response to ATRA by CD4^+^ and CD8^+^ Tregs and suggesting this possibility *in vivo*. In addition to blocking IL-6 for CD4^+^ Tregs expansion (79), deficiency in IL-27 signals enhances the proliferation of both CD4^+^ Tregs and CD8^+^ Tregs, suppressing the GVHD process (80). Moreover, coadministration of anti-IL-27 and resiquimod, agonists of Toll-like receptor 7, increases the number of CD4^+^ and CD8^+^ Tregs and alleviates GVHD (81). Nevertheless, IL-27-primed CD4^+^ Tregs show immunoregulatory properties to provide a protective role in GVHD (82). IL-27 acts on multiple cells, including proinflammatory and regulatory cells and the final results depend on the overwhelming mechanism. It also has been speculated that IL-27 exerts diverse effects in different stages of the GVHD process. In addition, transient anti-CD45RC treatment appears to induce CD45^hi^ T cell apoptosis and preserve CD8^+^CD45^low/-^ Tregs and CD4^+^CD45^low/-^ Tregs, enhancing regulatory ability (83). Immunoglobulin-like transcript 3 (ILT3) is an inhibitory receptor expressed on antigen-presenting cells (APCs), and some studies have identified the effect of ILT3-Fc in reducing GVHD, which may be due to induction of CD8^+^ Tregs (84). Inhibition of Cdk2 inactivates EZH2 and induces epigenetic regulation of Foxp3, leading to more CD8^+^ Tregs generation and GVHD prevention (85). These results reveal an unexpected mechanism by which Cdk2 inhibitors induce CD8^+^ Tregs. From another point of view, this is a great attempt to change the epigenetic characteristics of CD8^+^ Tregs.

### 5.2 Strengthening Stabilization

Pacritinib, a blocking agent of Janus kinase 2 (JAK2), has been reported to prevent GVHD while preserving the GVL effects (86). CD4^+^ Tregs generation is restrained by JAK2 signals, and the same effect on CD8^+^Foxp3^+^ Tregs indicates that blockade of JKA2 signals in CD8^+^Foxp3^+^ Tregs may effectively alleviate GVHD while preserving the GVL effects (22). Regarding mechanisms, blocking JAK2 signaling enhances the stability of CD8^+^Foxp3^+^ Tregs with upregulated expression of CD25, and even generates more CD4^+^ and CD8^+^ Tregs, which correlates with higher expression of neuropilin-1, a marker of Tregs, as well as demethylation of CNS2. An analogous mechanism has been reported for vitamin C (Vit-c), which exerts an effect on CD4^+^ Tregs (87), and the CNS2 region plays a significant role in expression of Foxp3 (88). However, it remains unclear whether Vit-c alleviates GVHD by affecting CD8^+^Foxp3^+^ Tregs. Vit-c as the cofactor of TET enzymes treatment can maintain demethylation of CNS2, and stabilize the expression of Foxp3 in CD8^+^ Tregs, thus promoting expression of CD25, Nrp1, Helios, CTLA-4 and PD-1 *in vitro*, preventing GVHD and maintaining the GVL effects (23). Indeed, Bim expression is able to impact Tregs by restraining proliferation and regulating apoptosis (89), which also contributes to imbalance between Tregs and effector T cells (90). Recent studies have found that Bim deficiency largely prolongs the survival of CD8^+^ Tregs, thus enhancing protection from GVHD (18). Notably, it is very different from earlier studies aiming at the stability of Foxp3.

### 5.3 Optimizing Function

With the maturation of immune cell-based targeting therapy technology, chimeric antigen receptor-redirected T cells (CAR-T) have been successfully used for the treatment of hematological malignancies, with curative effects (91, 92). HLA-A2-specific CAR CD4^+^ Tregs have greater regulatory GVHD abilities than polyclonal CD4^+^ Tregs (93), and accordingly, HLA-A2-specific CAR CD8^+^ Tregs are reported to prevent GVHD more effectively (94). However, there have been no studies to date demonstrating the influence on general immunity and the GVL effects. Engineered Tregs (eTregs) might offer an effective means to obtaining sufficient Tregs in the clinic. Much evidence shows that CD8^+^ eTregs derived from lentiviral transfection coexpressing Helios and Foxp3 effectively alleviate GVHD in a manner slightly superior to that of CD4^+^ eTregs (95). Furthermore, different isoforms of Helios show disparate influences on CD8^+^ and CD4^+^ Tregs, which should be taken into consideration in future studies.

## 6 Conclusion and Prospects

The ability to prevent GVHD without weakening GVL effects renders CD8^+^ Tregs superior to CD4^+^ Tregs, including enhancing GVL effects. Inducing CD8^+^ Tregs may ensure an adequate cell source. Although there is no doubt that CD8^+^ Tregs will be applied extensively for treating GVHD in the clinic, some obstacles and unsolved mechanisms remain to be explored. First, CD8^+^ Tregs are only present for a short time in GVHD, and such instability strongly affects application. Thus, we need feasible methods to stabilize phenotypes and regulatory abilities or to block factors that contribute to CD8^+^ Tregs conversion. Second, most CD8^+^ Tregs do not express Foxp3, and the exact function of Foxp3 in CD8^+^ Tregs remains unclear. Hence, other specific markers for CD8^+^ Tregs need to be identified to improve and facilitate their induction and isolation to achieve the best treatment in the clinic. Third, CD8^+^ Tregs can produce several inflammatory cytokines, such as IFN-γ, IL-2 and TNF-α (26, 28, 29, 49), which indicates that GVHD risk should be taken into consideration and that ensuring safe therapy needs more study. Optimal regulatory cell design may result in an ideal outcome, such as the combination of CD8^+^ and CD4^+^ Tregs exerting ameliorative results. In addition, microRNAs participate in regulating CD8^+^ Tregs function by regulating cytokine secretion or transcription factor expression; for example, miR-27b-3p and miR-340-5p negatively regulate the IL-10 level, and miR-330-3p acts on TGF-β expression (96). Foxp3 and CTAL-4 are negatively regulated by miR-335, miR-9 and miR-155 (97). Overall, the effect of microRNAs on CD8^+^ Tregs may influence the development of GVHD, and more evidence is needed.

For GVHD treatment, more studies are needed to identify different effects of drugs on CD8^+^ Tregs to improve outcomes of GVHD, because of the clearance or inhibition of these cells by immunosuppressive agents. For instance, cyclosporine (28) and cyclophosphamide suppress the generation and function of CD8^+^ Tregs (98). However, CD8^+^CD28^-^ Tregs are not influenced by methylprednisolone (58), which indicates their superiority in the combined application of CD8^+^CD28^-^ Tregs and glucocorticoids for GVHD. In addition, extracorporeal photopheresis (ECP), which is superior to other strategies because of the lack of bioactive materials, has been adopted to cure GVHD in the clinic, as ECP enhances the generation of both CD4^+^ and CD8^+^ Tregs (99). Nonetheless, the detailed mechanisms need to be elucidated.

In addition to immunoregulation to alleviate GVHD, how to exert powerful GVL effects remains another challenge for the development of cellular therapy technology. CAR CD8^+^ Tregs effectively alleviate GVHD, making it possible to achieve efficient GVL effects *via* CAR-T technology. However, immunosuppression or combination therapy may restrict CD8^+^ Tregs *in vivo*, and effective methods need to be identified for CD8^+^ Tregs application. Methods to eradicate such restriction may avoid tumor recurrence. In general, we should realize that the most abundant CD8^+^ Tregs are generated by autologous CD8^+^ T cells, and it is the key to constructing a suitable microenvironment *in vivo*.

Other approaches to strengthen immunoregulation capacity as well as novel solutions may be found in the interaction between CD8^+^ Tregs and other immune cells; for example, the regulatory function of mesenchymal stem cells (MSCs) is related to both CD4^+^ and CD8^+^ Tregs (100). In a human study of refractory cGVHD, MSC infusion induced a higher proportion (101) and enhanced the regulatory capacities of CD8^+^ Tregs, subsequently suppressing the proliferation and activation of CD4^+^ effector T cells and effectively alleviating cGVHD (102). Therefore, MSC-primed CD8^+^ Tregs may have much more potential, and other cell types have been proven to influence CD8^+^ Tregs function. Deficiency in liver kinase B1 (Lka1) in DCs greatly enhances the generation of CD8^+^ Tregs while maintaining high levels of Nrp1 and Helios, which indicates that the cells originate from the thymus (103). Lka1 may be a limitation for CD8^+^ Tregs, and therapy targeting Lka1 will overcome the intrinsic defect in the generation of CD8^+^ Tregs in GVHD. Moreover, human CD36^hi^ monocytes induce CD8^+^ Tregs *in vitro* and ameliorate GVHD by suppressing T cell proliferation, which also indicates that monocytes may assist in regulatory functions (104). However, the underlying mechanisms of cellular interactions *in vivo* need to be further investigated.

Taken together, we believe that CD8^+^ Tregs are powerful players in immune regulation and should be developed further through deeper and more systematic studies. Although much work is needed, all advances will provide understanding of CD8^+^ Tregs and optimization for CD8^+^ Tregs to ensure better outcomes for cell therapy in transplantation.

## Author Contributions

The manuscript was conceptualized by XZ and SY. WW wrote the majority of the manuscript, and XW and TH cowrote the manuscript. The figures were designed by WW and drawn with XW and RW. YD and QG produced the tables. All authors contributed to the article and approved the submitted version.

## Funding

This work was supported by the National Natural Science Foundation of China International Exchange and Cooperation Key Project (Grant No. 82020108004), National Center for Clinical Medicine Research on Blood System Diseases 2020 Open Project (Key Project) (Grant No. 2020ZKZC02), National Key Research Program “Stem Cell and Translational Research” Key Special Project (Grant No. 2017YFA0105502).

## Conflict of Interest

The authors declare that the research was conducted in the absence of any commercial or financial relationships that could be construed as a potential conflict of interest.

## Publisher’s Note

All claims expressed in this article are solely those of the authors and do not necessarily represent those of their affiliated organizations, or those of the publisher, the editors and the reviewers. Any product that may be evaluated in this article, or claim that may be made by its manufacturer, is not guaranteed or endorsed by the publisher.
